# Association of the Middle Accessory Cerebral Artery With Bihemispheric Anterior Cerebral Artery and Median Artery of the Corpus Callosum

**DOI:** 10.7759/cureus.86733

**Published:** 2025-06-25

**Authors:** Gervith Reyes Soto, Carlos Castillo-Rangel, Luis Delgado-Reyes, Danil Nurmukhametov, Carlos Ernesto López Lara, Julio Cesar Pérez Cruz, Andreina Rosario Rosario, Manuel De Jesus Encarnacion Ramirez

**Affiliations:** 1 Neurosurgical Oncology, Mexico National Cancer Institute, Tlalpan, MEX; 2 Neurosurgery, Servicie of the 1ro de Octubre Hospital of the Instituto de Seguridad y Servicios Sociales de los Trabajadores del Estado (ISSSTE), Mexico City, MEX; 3 Anatomy, Faculty of Medicine, National Autonomous University of Mexico, Mexico City, MEX; 4 Medicine, I.M. Sechenov First Moscow State Medical University, Medical Sechenov Pre-university, Moscow, RUS; 5 Neurosurgery, Peoples’ Friendship University of Russia, Moscow, RUS; 6 Anatomical Techniques and Teaching Materials Laboratory, National Institute, Superior Medicine School, Mexico City, MEX; 7 Medicine, Autonomous University of Santo Domingo (UASD), Santo Domingo, DOM; 8 Human Anatomy and Histology, N.V. Sklifosovsky Institute of Clinical Medicine, Moscow, RUS

**Keywords:** bihemispheric anterior cerebral artery, corpus callosum, hipoplasic, latex injection, neuroanatomy

## Abstract

As part of a microsurgical anatomy study of the recurrent artery of Heubner (RAH), we identified three rare vascular variants in a 45-year-old female cadaver: the right middle accessory cerebral artery (MACA), bihemispheric anterior cerebral artery (Bihem-ACA), and median artery of the corpus callosum (MACC). These anomalies were documented through meticulous dissection and detailed morphometric analysis, underscoring the value of cadaveric studies in elucidating complex cerebral vascular anatomy. The specimen was obtained within 24 hours postmortem. The cerebral arteries were injected with red latex and fixed using a standard protocol: initial perfusion with formaldehyde, followed by immersion in 10% formalin for a total fixation period of two months. Dissection was performed under a Carl Zeiss OPMI™ surgical microscope (Carl Zeiss Meditec AG, Jena, Germany) at 6x-40x magnification. Morphometric measurements were taken using a digital Mitutoyo vernier caliper (Mitutoyo South Asia Pvt. Ltd., New Delhi, India) with 0.01 mm resolution. During dissection, the right MACA was observed originating from the anterior communicating artery (ACoA), proximal to the junction of the A1 segments. It coursed posteriorly, running parallel to the right pre-communicating segment (A1) of the ACA and the sphenoidal (M1) segment of the middle cerebral artery (MCA). This artery measured 67 mm in length and 0.6 mm in diameter, with two perforating branches arising approximately 25 mm from its origin. The right A1 (d-A1) segment was dominant, measuring 14 mm in length, with a 1.5 mm diameter and an 8 mm outer perimeter, continuing distally as a single Bihem-ACA. This trunk extended 27 mm before bifurcating, with a proximal diameter of 1.7 mm and an outer perimeter of 9 mm. In contrast, the left A1 segment was hypoplastic, measuring 16.72 mm in length, 1 mm in diameter, and 5.5 mm in outer perimeter. The MACC originated from the left A3 segment of the ACA and measured 2.1 mm in diameter and 40 mm in length. It gave rise to two right cingulate-callosal branches (0.5 mm and 0.4 mm in diameter, respectively) directed toward the callosal sulcus and cingulate gyrus. These findings highlight an unusual combination of vascular variants not commonly observed together in a single individual. Recognizing such configurations is crucial for surgical planning, particularly in procedures involving the interhemispheric fissure, anterior communicating complex, and corpus callosum. Enhanced anatomical knowledge of these rare variants supports safer neurosurgical interventions and improves intraoperative decision-making in cerebrovascular procedures.

## Introduction

The cerebral vasculature is highly complex and variable, posing significant challenges and opportunities for neurosurgeons and radiologists. Among its critical components, the anterior cerebral artery (ACA) and its branches are vital for supplying blood to the medial and superior parts of the frontal lobes, the corpus callosum, and the cingulate gyrus. Variations in ACA anatomy can significantly impact surgical planning and the management of cerebrovascular diseases [1,2].

The ACA typically arises from the bifurcation of the internal carotid artery (ICA), near the base of the Sylvian fissure and lateral to the optic chiasm. From there, it travels anteromedially, passing over the optic nerve or chiasm, and enters the interhemispheric fissure. Within this region, it connects with the opposite ACA through the anterior communicating artery (ACoA), forming what is known as the A1 segment [3,4]. Beyond this connection, the ACA continues and divides into multiple parts, including the pericallosal artery, which is further subdivided into four segments: A2 through A5 [5, 6].

The middle cerebral artery (MCA), initially described by Lautard in 1892 and later renamed by Looten in 1906, also exhibits significant anatomical variations. These include accessory arteries and duplications, which can complicate diagnostic imaging and surgical intervention [7, 8]. The ACoA located in the interhemispheric fissure, has clinically significant variants such as the accessory MCA (a-MCA) and the duplicated MCA (d-MCA), impacting cerebral blood flow and the potential for aneurysm formation [9, 10].

We present a rare instance of combined vascular anomalies involving the right middle accessory cerebral artery (MACA), bihemispheric anterior cerebral artery (Bihem-ACA), and median artery of the corpus callosum (MACC). These findings were observed during the microsurgical anatomical study of the recurrent artery of Heubner (RAH). The specimen, a 45-year-old female cadaver, revealed these rare vascular formations, each meticulously measured and documented. The right MACA was noted to originate from the ACoA, running parallel to the pre-communicant segment of the right ACA and the sphenoidal segment of the right MCA, with significant morphometric details recorded.

Our findings underscore the importance of detailed anatomical studies and cadaveric dissections in accurately identifying and understanding these variations. Cadaveric studies are indispensable for visualizing the complex anatomy of cerebral arteries and planning neurosurgical interventions [11, 12]. By providing hands-on, three-dimensional perspectives, cadaveric studies allow clinicians to map out vascular anomalies and tailor their surgical approaches accordingly, thereby improving patient outcomes [13, 14].

## Case presentation

Specimen preparation

A brain specimen was obtained from the Microsurgical Anatomy of the Central Nervous System Laboratory at the Faculty of Medicine, National Autonomous University of Mexico (UNAM), Mexico City, Mexico. The donor was a 45-year-old female cadaver with a postmortem interval of less than 24 hours and no history of neurological disease.

Vascular injection and fixation protocol

To preserve the cerebral vascular architecture for microanatomical analysis, a standardized seven-step protocol was followed.

Extraction

The brain was carefully removed from the cranial cavity, ensuring minimal disruption to the vasculature. Cranial nerves, arteries, veins, and venous sinuses were transected close to the skull base. Key vascular structures, including the ICAs, vertebral arteries (V4 segment), and major venous sinuses, were preserved where possible for accurate anatomical correlation.

Vascular Washout

The right ICA and right vertebral artery were catheterized using 5 French (Fr) catheters. The contralateral vessels were ligated. A saline solution was gently perfused to remove residual blood and thrombi, preventing obstruction of vascular injection. Sites of leakage, particularly near the internal auditory artery, were identified and sealed to ensure complete system perfusion.

Perfusion Fixation

To achieve optimal tissue preservation and maintain vessel integrity, 15 mL of pure formaldehyde was perfused through both catheterized arteries for five minutes. The brain was then immersed in 3 liters of 10% buffered formalin for 15 minutes, followed by an additional 1-liter perfusion through the ICA while maintaining an open vertebral artery. This protocol ensured thorough fixation of intracerebral and extracerebral vessels.

Latex Injection

After fixation, the brain was removed and prepared for contrast injection. A white latex compound (Poliformas Plásticas®, Mexico City, Mexico) mixed with carmine 319 acrylic paint (Politec®, Mexico City, Mexico) was used to enhance vascular visibility. Fifteen milliliters of the mixture were injected through the vertebral artery and 20 milliliters through the ICA. Excess latex was carefully washed off to prevent contamination of the leptomeninges.

Post-injection Immersion Fixation

The latex-injected brain was submerged in fresh 10% formalin for 24 hours. The solution was then replaced, and the specimen remained immersed for an additional two-month fixation period to ensure complete tissue stabilization prior to dissection.

Final Preservation

After fixation, the brain was rinsed for 24 hours under running water to remove formalin residues and subsequently preserved in a 60% isopropyl alcohol solution to maintain tissue pliability and color contrast during dissection.

Dissection and morphometric analysis

Microsurgical dissection was performed under a Carl Zeiss OPMI™ surgical microscope (Carl Zeiss Meditec AG, Jena, Germany) at magnifications ranging from 6x to 40x. Instruments included microsurgical scissors and No. 3 watchmaker’s forceps. The cerebral arterial system was carefully isolated, and each identified vessel variant was documented with high-resolution digital photography.

Morphometric measurements such as arterial diameter, length, and branching angles were obtained using a Mitutoyo digital Vernier caliper (Model CD-8” CX; Mitutoyo Corporation, Kanagawa, Japan) with a resolution of 0.01 mm. Measurements were taken in situ, ensuring anatomical context relative to cerebral landmarks such as the optic chiasm, corpus callosum, anterior perforated substance, and interhemispheric fissure.

Measurements

Measurements of the artery's diameter, length, and distances from key anatomical landmarks used for dissection, such as the ICA, anterior perforated substance, and ACoA, were taken using a Mitutoyo digital Vernier caliper (Model CD-8” CX, resolution of 0.0005”/0.01mm, Mitutoyo South Asia Pvt. Ltd., New Delhi, India). Specific measurements included diameter at origin, length from origin to bifurcation, and diameters of medial and lateral branches.

Distance From ICA Origin to the Posterior Clinoid Process and Morphometric Analysis

The number of perforating branches and their diameters were recorded. Detailed morphometric descriptions of segments A1, a-MCA, Bihem-ACA, right A1 (d-A1), right M1 (d-M1), right M2 bifurcation (d-M2B), right ICA (d-ICA), right olfactory bulb (d-OB), right orbitofrontal artery (d-OFA), left A1 (i-A1), RAH, fronto-basal trunk (FBT), and MACC were performed.

Data recording and documentation

High-resolution digital photographs were captured using an Olympus™ μ DIGITAL 800 8.0-megapixel camera (Olympus Corporation, Tokyo, Japan). All findings were meticulously recorded and compared with existing literature to highlight variations and clinical implications [8]. 

Results

**Figure 1:**
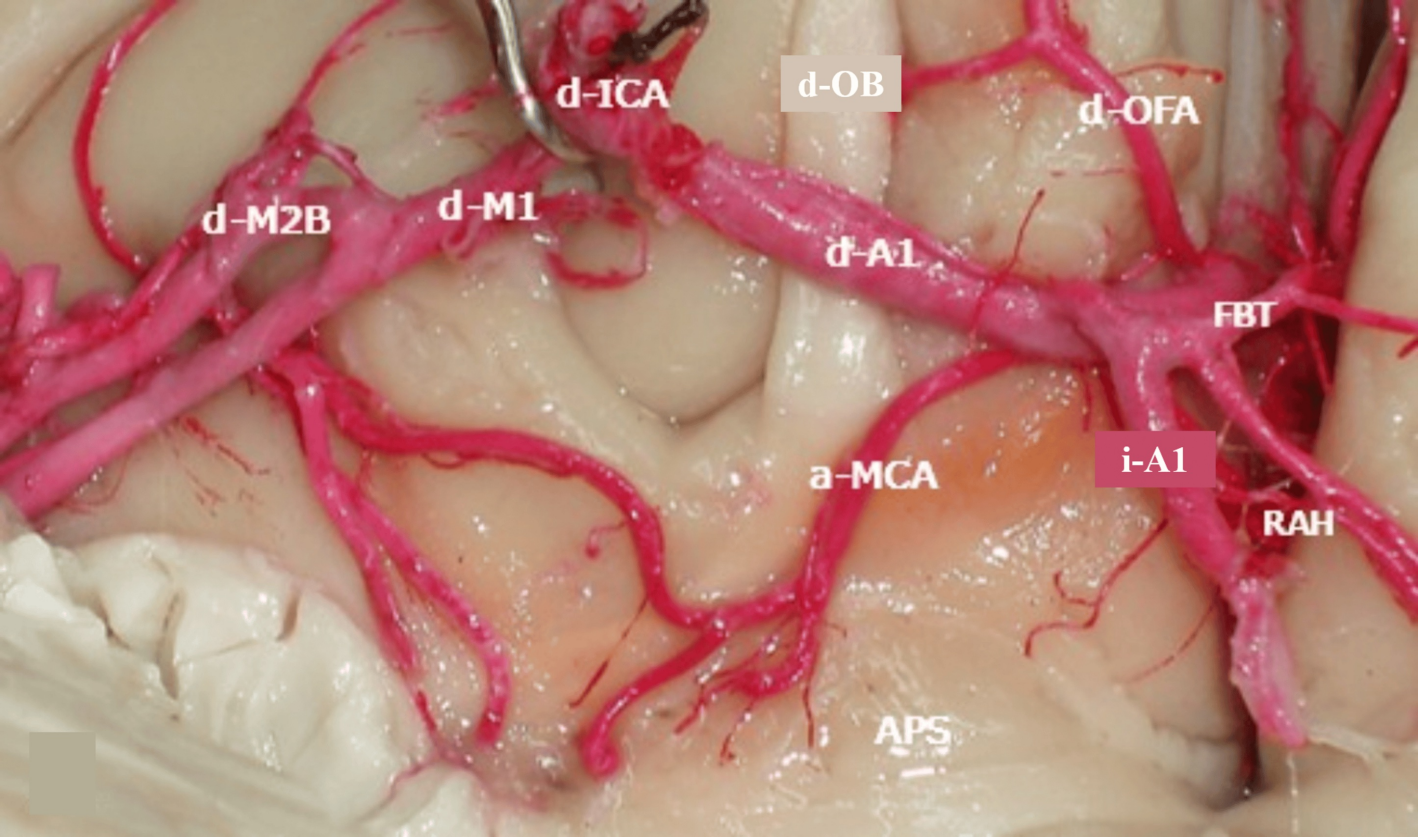
Microscopy of the accessory middle cerebral artery (a-MCA) APS: anterior perforated substance; d-A1: right A1; d-M1: right M1; d-M2B: right M2 bifurcation; d-ICA: right internal carotid artery; d-OB: right olfactory bulb; d-OFA: right orbitofrontal artery; i-A1: left A1; RAH: recurrent artery of Heubner; FBT: fronto-basal trunk

**Figure 2:**
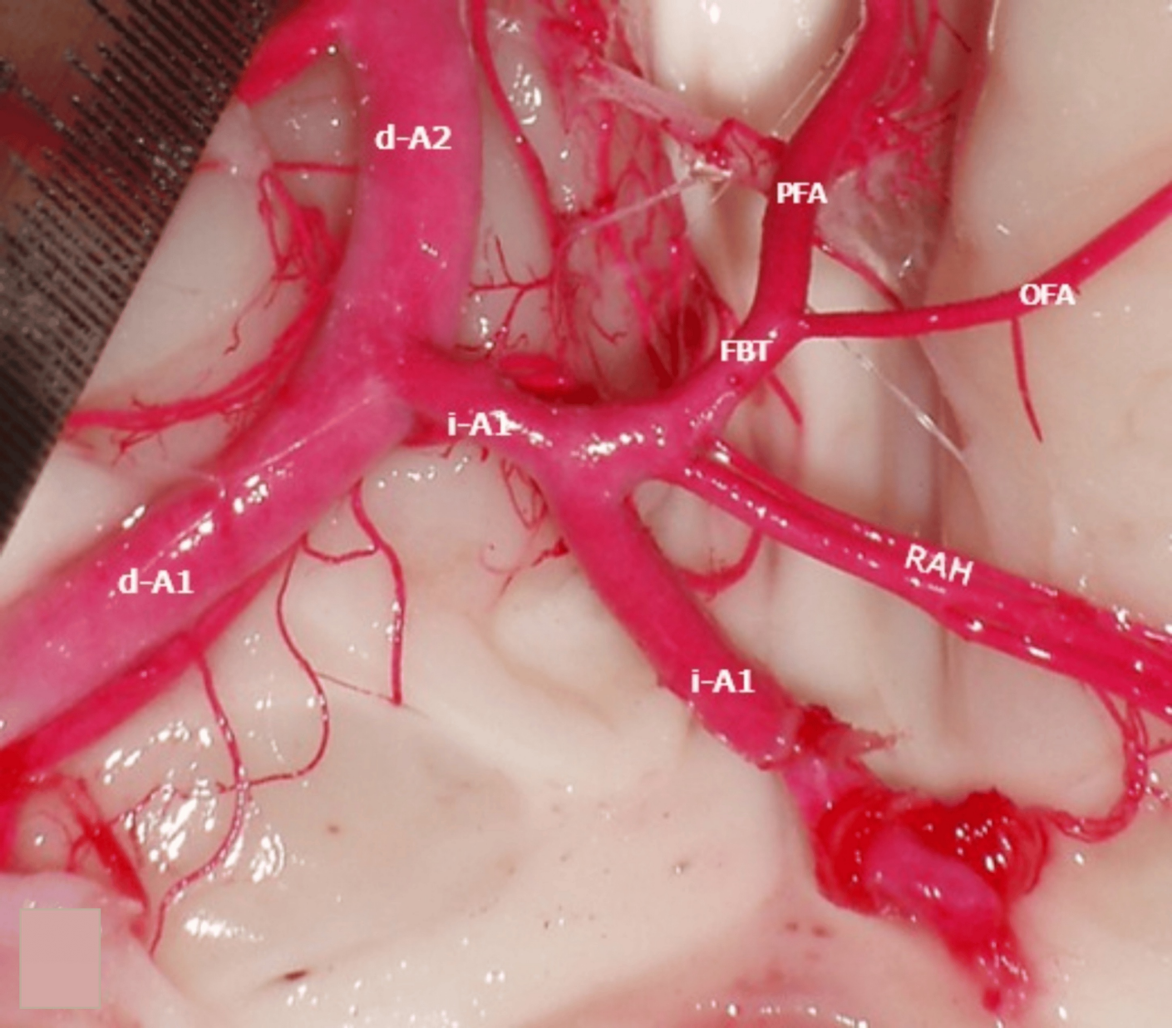
A1-A2 junction d-a1: right A1; i-A1: left A1; RAH: recurrent artery of Heubner; i-A2: left A2; d-A2: right A2; FBT: fronto-basal trunk; OFA: orbitofrontal artery; FPA: frontopolar artery

**Figure 3:**
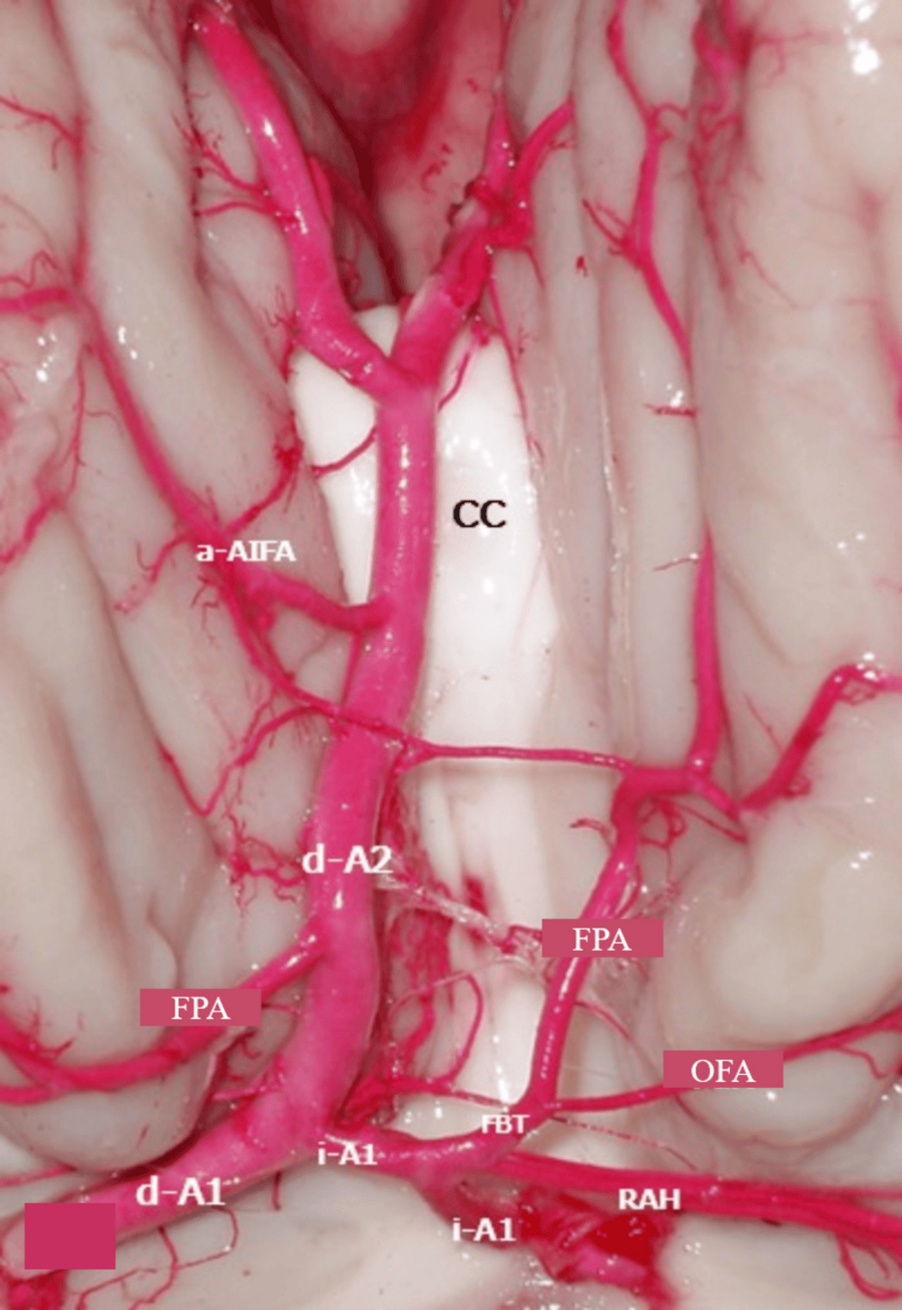
Microphotograph of the anterior cerebral artery (ACA) segments d-a1: right A1; i-A1: left A1; i-A2: left A2; d-A2: right A2; CC: corpus callosum; RAH: recurrent artery of Heubner; FBT: fronto-basal trunk; OFA: orbitofrontal artery; FPA: frontopolar artery; a-AIFA: anterior internal frontal artery

**Figure 4:**
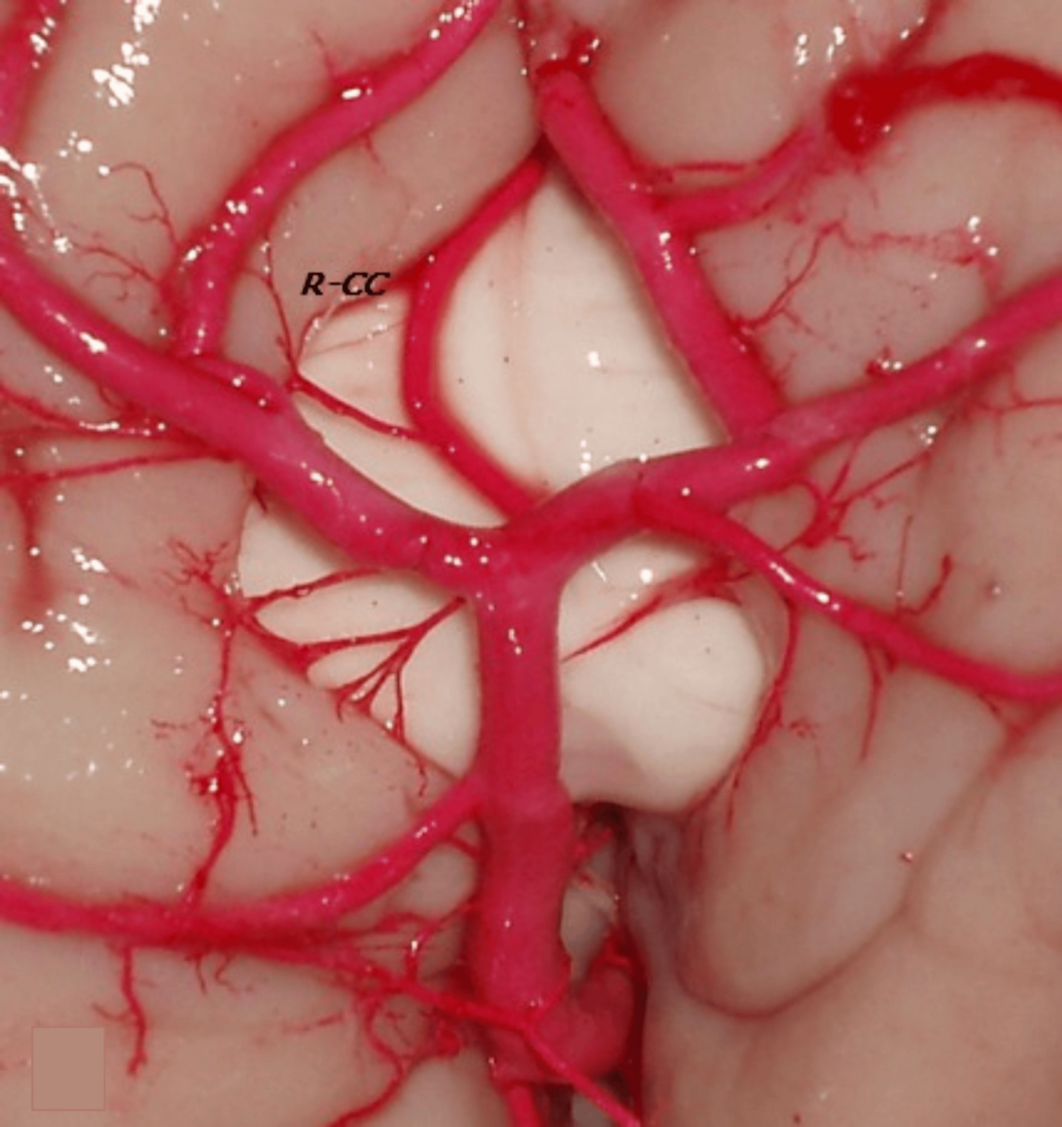
The A3 segment bilaterally gave rise to the callosomarginal arteries (CaM), with the right CaM having a diameter of 4.3 mm and the left CaM 3.5 mm. The proximal pericallosal artery (A2 segment) measured 2.0 mm, and the mid pericallosal artery (A3–A4 segments) measured 1.5 mm. Branches from the right pericallosal segment reached the frontal, parietal, and occipital lobes on the medial surface bilaterally. R-CC: right corpus callosum

A 45-year-old female brain specimen weighing 1,250 grams was obtained. The A1 segment of the ACA originated bilaterally from the medial face of the ICA, ventral to the olfactory trigone. After a brief horizontal course, it deviated anteromedially, entering rostrally to the chiasm towards the interhemispheric fissure, where it joined its counterpart to form a single arterial trunk (unique A2). The right A1 segment (A1-d) was dominant, with a length of 14 mm, an outer perimeter of 8 mm, and a diameter of 1.5 mm. The left A1 segment (A1-I) was hypoplastic with a length of 16.72 mm, a diameter of 1 mm, and an outer perimeter of 5.5 mm (Figures 1-4).

The perforating branches originating from the lateral edge of the A1 segment supplied the rostrum of the corpus callosum, lamina terminalis, middle part of the anterior commissure, optic chiasm, and paraolfactory area. The number of perforating branches in d-A1 was 12, with an average diameter of 0.005-1.90 mm, while i-A1 had 18 branches with an average diameter of 0.006-1.10 mm.

The duplicated left RAH originated from the lateral edge of A2, directed towards the anterior perforated substance, with a diameter of 1.90 mm and a length of 30.5 mm. A common trunk and bifurcation were observed 9 mm from its origin. On the right side, a-MCA and d-MCA originated at the junction of both precommunicating segments (A1) of the ACA, running retrogradely parallel to the d-A1 segment and then to the sphenoidal segment (M1) of the d-MCA, towards the orbital surface of the frontal lobe. Its length was 67 mm and its diameter was 0.6 mm. During its course, two branches towards the anterior perforated substance were observed 25 mm from its origin, measuring 0.3 mm and 0.4 mm, respectively (Figure 1).

The ACoA was not found; instead, a hypoplastic i-A1 was observed with a diameter of 4 mm and a length of 16.72 mm from the bifurcation of the left carotid artery to the junction with the medial edge of A1-d, where its course ended before giving rise to a common trunk for the left RAH and the left fronto-basal artery (FBA). The FBA originated from the left orbito-frontal artery (i-OFA) and the left fronto-polar artery (i-FPA), the latter irrigating the medial surface of the frontal lobe and giving a branch to the medial surface of the contralateral frontal lobe. Its length from the origin of the FBA to d-A1 was 8 mm with a diameter of 1 mm. The segment from the origin of the FBA to the junction of i-A1 with d-A1 presented three perforating branches with a diameter of 0.7 mm (Figure 2).

The unique A2 segment began after the union of left A1 (i-A1) to the medial edge of the dominant d-A1, at which point it was named the right Bihem-ACA. It headed towards the interhemispheric fissure, entering the cistern of the corpus callosum, where it coursed rostrally to the lamina terminalis, continuing as segment A3, anterior to the genu of the corpus callosum. Segment A3 of the Bihem-ACA bifurcated into a right A3 (d-A3) and a left A3 (i-A3), with a length of 30 mm and a diameter of 1.5 mm, later giving rise to the pericallosal artery and the callosomarginal artery (CaM) located on the trunk of the CC. The right Bihem-ACA had a length of 27 mm before bifurcation, with an origin diameter of 1.7 mm (Figure 3).

From its i-A3 segment, the right Bihem-ACA gave rise to the MACC, with an origin diameter of 2.1 mm and a length of 40 mm, which originated two right cingulate-callosal branches with diameters of 0.5 mm and 0.4 mm, directed towards the callosal sulcus and cingulate gyrus. The A3 segment bilaterally originated the CaMs, with a diameter of 4.3 mm for the right CaM and 3.5 mm for the left CaM (and the diameters of the pericallosal arteries) (Figure 4). The branches derived from the right pericallosal segment reached the medial surface of the frontal, parietal, and occipital lobes bilaterally.

## Discussion

The ACA, a terminal branch of the ICA, plays a pivotal role in supplying the medial and superior portions of the frontal lobes, the corpus callosum, and the cingulate gyrus. Anatomically, the ACA courses anterior to the optic chiasm and olfactory trigone, entering the interhemispheric fissure after joining its contralateral counterpart via the ACoA [15,16]. It then follows the curvature of the corpus callosum, terminating at its splenium [17].

In our study, we documented three rare vascular anomalies: a right MACA, a Bihem-ACA, and a MACC. These findings provide a unique anatomical arrangement not previously described in a single specimen and have notable implications for cerebrovascular surgery and neuroimaging.

The ACA’s cortical branches-orbital, frontal, and parietal-exhibit a well-documented degree of variability, supplying key regions including the olfactory tract, cingulate gyrus, and precuneus [18]. This variation is largely attributed to embryological development from a single interhemispheric vascular plexus [6,18,19,20]. The presence of the MACC in our specimen exemplifies this embryological origin, representing a persistent median artery that may arise when longitudinal channels fail to regress during development. Comparative literature suggests that MACC has been identified in isolated reports but rarely in conjunction with other anomalies [10,20].

Our observation of a Bihem-ACA, with a dominant d-A1 segment and a hypoplastic i-A1, aligns with previously reported morphological variants. Brandt’s morphometric study identified such asymmetry in up to 21.5% of cases, with left-sided hypoplasia being more frequent [21,22]. However, the combination of a Bihem-ACA continuing as a single A2 segment supplying both hemispheres, alongside a concurrent MACA and MACC, distinguishes this case from prior literature and warrants further comparative anatomical analysis.

The MACA in our case originated from the ACoA region and coursed parallel to the M1 segment of the MCA. According to Yasargil and Crompton, such arteries are typically classified as a-MCA when arising from the ACA and as d-MCAs when originating from the ICA [23,24]. The MACA in our study conformed to the a-MCA type, with a distribution overlapping that of the MCA territory. Notably, it also gave off perforating branches to the anterior perforated substance, a feature that may increase the risk of intraoperative injury if unrecognized.

Although our findings were based on a single cadaveric specimen, they highlight critical anatomical nuances relevant to aneurysm surgery, stroke management, and tumor resections involving the corpus callosum and interhemispheric fissure. For example, the presence of a Bihem-ACA can influence collateral circulation dynamics, while the MACC may complicate access to pericallosal or CaMs during surgery. These observations underscore the importance of preoperative vascular imaging and suggest the potential utility of 3D printing technologies to create individualized surgical planning models, particularly when such rare variants are suspected.

Importance of the study

Understanding ACA variations is crucial for neurosurgeons and radiologists. The ACA supplies critical brain areas, including the medial and superior parts of the frontal lobes, the corpus callosum, and the cingulate gyrus. Anatomical variations can significantly influence surgical planning and the management of cerebrovascular diseases, aiding in avoiding surgical complications and optimizing patient outcomes. Understanding ACA variations is essential for optimizing surgical outcomes in cerebrovascular procedures, and recognizing the anatomical and functional complexity of the region is equally critical, given its key role in motor coordination and its implications in neurological disorders such as hypertrophic olivary degeneration. This study emphasizes the frequency and types of ACA variations, underscoring the necessity for precise preoperative imaging and careful intraoperative navigation [21,23,25].

Anatomical variations and clinical implications

The ACA exhibits several anatomical variants that can impact cerebral pathology presentation and management. Common variations include the absence, bifurcation, or hypoplasia of the A1 segment and the presence of accessory or azygos ACAs. These can alter cerebral hemodynamics, increasing the risk of aneurysm formation at the ACoA complex [15,18,20,22,25]. Kimura et al. [9] confirmed the association between altered hemodynamics due to A1 segment asymmetry and increased aneurysm risk.

For instance, a hypoplastic A1 segment can lead to compensatory increased blood flow through the contralateral ACA, predisposing to aneurysm formation due to altered hemodynamic stress [26]. The presence of an azygos ACA, where a single ACA supplies both hemispheres, Kihira et al. [6] documented the anatomical and surgical implications of this rare variant, while Fujiwara et al. [27] noted that its presence complicates interhemispheric surgical approaches, especially in tumor resections of the corpus callosum. [28].

Variations and neurosurgical approaches

In aneurysm contexts, ACA anatomical variations are particularly significant. The anterior communicating artery is a common aneurysm site, and variations such as hypoplasia or aplasia of the A1 segment affect both presentation and surgical or endovascular treatment approaches. Preoperative recognition of these variations through advanced imaging is essential to minimize complications during aneurysm clipping or coiling [ 28,29].

For glioma treatment, especially in the frontal lobe or corpus callosum, knowledge of ACA variations is vital. Surgical resection of gliomas requires precise navigation to avoid damaging vital vascular structures. Variations like accessory ACA or azygos ACA pose challenges in tumor resection while preserving adequate brain blood supply. Intraoperative mapping and neuro-navigation systems incorporating detailed vascular anatomy help achieve maximal tumor resection with minimal neurological deficits [30,31].

Implications for stroke and other cerebrovascular diseases

The ischemic damage in the ACA territory may extend via diaschisis to deeper structures, including the brainstem [32]. Specifically, stroke-induced disruption of supratentorial pathways may lead to secondary degeneration in downstream networks such as the Guillain-Mollaret triangle, a triangular circuit connecting the red nucleus, inferior olive, and dentate nucleus. When affected, this can result in hypertrophic olivary degeneration and movement disorders, such as palatal myoclonus and Holmes tremor [33].

Enhancing preoperative planning

For neurosurgeons, detailed anatomical information from cerebral angiography is invaluable for preoperative planning. Understanding the exact location and configuration of vascular anomalies helps strategize surgical approaches, minimizing intraoperative risks and enhancing the likelihood of successful outcomes. For aneurysm repair, knowing the precise anatomy of the anterior communicating artery complex and its variations guides the surgical approach, reducing complication risks [34,35,36].

Risk assessment and management

Anatomical variations in cerebral vasculature significantly impact cerebrovascular disease risk profiles. Cerebral angiography aids early detection of these variations, enabling proactive risk assessment and management. For instance, individuals with hypoplastic A1 segments or azygos ACA may have increased aneurysm formation risks due to altered hemodynamics. Identifying these variations through angiography allows tailored surveillance and intervention strategies, potentially preventing catastrophic events [35,36].

Implications for endovascular procedures

In endovascular procedures like stenting and coiling, cerebral angiography is indispensable. It provides a roadmap for navigating complex vascular terrain, ensuring precisely targeted interventions. The ability to visualize small, otherwise occult branches, like the a-MCA or MACC, ensures effective and safe endovascular treatments, avoiding inadvertent damage to vital structures [34,37] Komiyama et al. highlighted the importance of identifying such variants in the anterior circulation, especially in the setting of aneurysms or arteriovenous malformations [13]. Accurate identification ensures safer and more effective endovascular treatment while minimizing iatrogenic complications.

Educational value and training

Cerebral angiography holds significant educational value for neurosurgeons and radiologists in training. Exposing trainees to anatomical variations enhances understanding and prepares them for diverse clinical scenarios. This hands-on experience with real-world anatomical diversity is crucial for developing skills and confidence to manage complex cerebrovascular conditions [38,39,40].

Embryological development and variability

The embryological development of the ACA as a single interhemispheric plexus differentiating into distinct branches results in wide-ranging vascular configurations, from typical patterns to rare anomalies like the Bihem-ACA or median callosal artery [12]. These variations underscore cerebral vascular anatomy complexity, necessitating individualized diagnostic and treatment approaches.

Integration of 3D printing in neurosurgical planning

3D printing technology has revolutionized neurosurgery, offering powerful tools for preoperative planning and patient-specific surgical simulations. Accurate, tangible models of the brain's vascular structures, including the rare ACA variations documented in this study, enable better visualization of complex anatomical relationships and more precise planning [40,41]. By creating 3D-printed replicas of these rare vascular formations, surgeons can practice and refine their techniques, enhancing their spatial awareness and technical skills before performing actual surgeries. This approach not only improves surgical outcomes but also minimizes the risk of complications. Additionally, 3D printing enhances educational opportunities, allowing trainees to interact with models representing anatomical variations [42,43]. This hands-on experience is crucial for developing the skills and confidence necessary for managing complex cerebrovascular conditions [44, 45].

Limitations of the study

Despite the valuable insights provided by this study on the rare vascular variants involving the right MCA, Bihem-ACA, and MACC, several limitations must be acknowledged.

Sample Size and Generalizability

Further studies involving larger and more diverse sample sizes are necessary to validate these findings and determine their prevalence across different demographic groups [3,46].

Methodological Constraints

The methodology employed, including the injection of red latex and fixation with 10% formalin, while standard, may introduce artifacts that could affect the anatomical measurements. The process of dissection under a surgical microscope, though precise, is still subject to human error. Additionally, the use of a digital vernier caliper for measurements, although accurate, might not capture the finest nuances in vascular dimensions, potentially leading to slight inaccuracies [3, 40].

Anatomical Variations

The study focuses on specific vascular variations within a single individual, which may not capture the full spectrum of anatomical diversity. Vascular anatomy is highly variable, and the presence of unique anomalies in one specimen might not reflect common patterns observed in the general population. This limitation underscores the need for more extensive studies to identify the range and frequency of these variations [47].

Lack of Functional Correlation

Another significant limitation is the lack of correlation with functional or clinical outcomes. While the anatomical descriptions are detailed, the study does not provide information on how these variations might impact cerebral function or predispose individuals to specific pathologies. Future studies should aim to correlate anatomical variations with clinical data to better understand their implications in neurological conditions such as aneurysms, gliomas, and strokes [34,48,49].

Lack of Comparative Analysis

One limitation of the study is the absence of a comparative analysis with other specimens or established reference standards. Incorporating such comparisons would offer a more meaningful context for interpreting these rare anatomical variations, helping to clarify how they differ from more common configurations. Comparative research could also shed light on whether these anomalies are isolated findings or part of a wider pattern of vascular variability and limited technological integration.

While the study provides a detailed anatomical account, it does not leverage advanced imaging technologies such as high-resolution MRI or CT angiography, which could offer more precise and comprehensive visualization of vascular structures. Incorporating these technologies in future studies would enhance the accuracy of anatomical descriptions and allow for non-invasive correlation with clinical conditions [49,50].

Potential impact on clinical practice

Despite these limitations, the findings of this study have important implications for clinical practice, particularly in neurosurgery and radiology. Understanding these rare anatomical variations can aid in preoperative planning, improve surgical outcomes, and reduce the risk of complications. However, the clinical relevance of these findings needs to be validated through further research involving larger cohorts and diverse populations [51, 52].

## Conclusions

A thorough examination of ACA anatomical variations offers valuable insights for clinical practice. These differences play a crucial role in how conditions like aneurysms, gliomas, strokes, and other cerebrovascular disorders are managed. Utilizing advanced imaging technologies alongside innovations like 3D printing, paired with a deep understanding of anatomical variations, is key to successful treatment planning and better patient outcomes. Moving forward, ongoing research should continue to investigate how these variations impact clinical care and work toward creating clear guidelines for managing them in different neurological scenarios.

Recognizing and understanding ACA anatomical differences is more than just an academic exercise; it’s essential for real-world applications. As this study shows, these variations can directly influence the success of neurosurgical procedures and the treatment of cerebrovascular conditions.
